# H6PD overexpression promotes ex vivo expansion of human cord blood hematopoietic stem cells

**DOI:** 10.1007/s12015-022-10352-w

**Published:** 2022-02-05

**Authors:** Yuting Jin, Qin Wang, Qingwei Ding, Chunxu Yao, Rongzhen Jiang, Bin Guo, Qingyou Meng

**Affiliations:** 1 Obstetric Intensive Care Center, The Institute of Obstetrics and Gynecology, Department of Obstetrics and Gynecology, Shanghai Jiao Tong University Affiliated Sixth People’s Hospital, Shanghai, 200233 China; 2 Department of Pathophysiology, Key Laboratory of Cell Differentiation and Apoptosis of Chinese Ministry of Education, Shanghai Jiao Tong University School of Medicine, Shanghai, 200025 China; 3 Department of Gynaecology and Obstetrics, the First People’s Hospital of Kunshan, KunshanJiangsu, 215300 China; 4 Department of Vascular Surgery, General Surgery Clinical Center, Shanghai General Hospital, Shanghai Jiao Tong University School of Medicine, Shanghai, China

## Abstract

**Supplementary Information:**

The online version contains supplementary material available at 10.1007/s12015-022-10352-w.

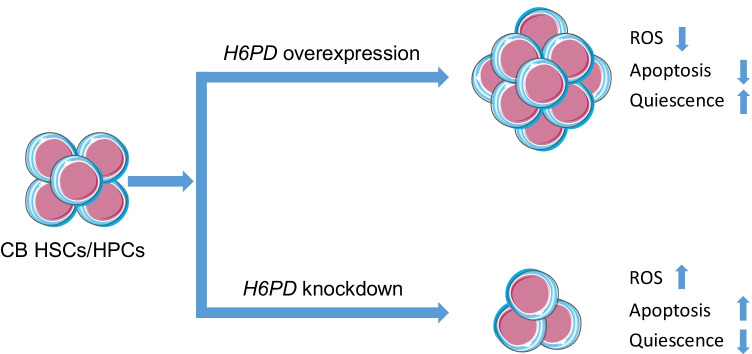

**To the Editor:**

Ex vivo expansion is one of the potential approaches to overcome the rarity of of hematopoietic stem cells (HSCs) for clinical application. Exploring the mechanism and regulation of ex vivo expansion of cord blood (CB) HSCs may facilitate the establishment of efficient ex vivo expansion system. Long-term HSCs with high repopulating capacity usually stay quiescent and mostly use glycolysis as the major metabolic approach[1, 2]. Due to the low mitochondrial metabolic activity, ROS level is kept lower in LT-HSCs compared with short-term (ST) HSCs or multipotent progenitors (MPPs)[1]. Ectopic accumulation of ROS impairs the quiescence and engrafting capacity of LT-HSCs by inducing cell differentiation, senescence and apoptosis[3]. NAPDH is a major intracellular reducing power, and protects intracellular components from ROS induced damage. The pentose phosphate pathway is an alternative glucose oxidizing pathway for the generation of NADPH, which is essential for reductive biosynthetic reactions[4]. Pentose phosphate pathway is a branch of glycolysis, and the role of key components of this pathway in HSC expansion has not been determined.

In order to check the role of pentose phosphate pathway in CB HSC expansion, we focused on the two types of rate limiting enzymes including H-form glucose-6-phosphate dehydrogenase (H6PD), and G-form glucose-6-phosphate dehydrogenase (G6PD). We found that G6PD was diffusely expressed in the cytoplasma, while H6PD was specifically expressed at ER (Supplementary Fig. 1A). Interestingly, *H6PD* OE significantly promoted ex vivo expansion of CD34^+^CD133^+^ADGRG1^+^ HSCs and CD34^+^CD133^+^ HPCs (Supplementary Fig. 1B-D). However, *G6PD* OE had no notable effect on ex vivo expansion of neither CD34^+^CD133^+^ADGRG1^+^ HSCs nor CD34^+^CD133^+^ HPCs (Supplementary Fig. 1B-D). To further examine the role of H6PD in CB HSC expansion, we performed knockdown experiments using CD34^+^ cells. Control shRNA or *H6PD* shRNA were transfected into CB CD34^+^ cells, and *H6PD* was efficiently knocked down by *H6PD* shRNA (Supplementary Fig. 2A). *H6PD* KD remarkably suppressed ex vivo expansion of CD34^+^CD133^+^ADGRG1^+^ HSCs and CD34^+^CD133^+^ HPCs(Supplementary Fig. 2B-D).

Next, to determine if *H6PD* OE expanded CB HSCs are functional in vivo, we did limiting dilution analysis (LDA) to calculate the number of SCID repopulating cells (SRCs) in control vector and *H6PD* OE CD34^+^ cells. Poisson distribution analysis revealed an SRC frequency of 1:2,929 in control vector transfected CB CD34^+^ cells and 1:1009 in *H6PD* OE CB CD34^+^ cells, suggesting the presence of 341 SRCs and 991 SRCs (2.9-fold increase) in 1 × 10^6^ ex-vivo cultured CD34^+^ cells (Fig. 1A-C). These data suggests that *H6PD* OE promotes ex vivo expansion of functional HSCs.

**Fig. 1:**
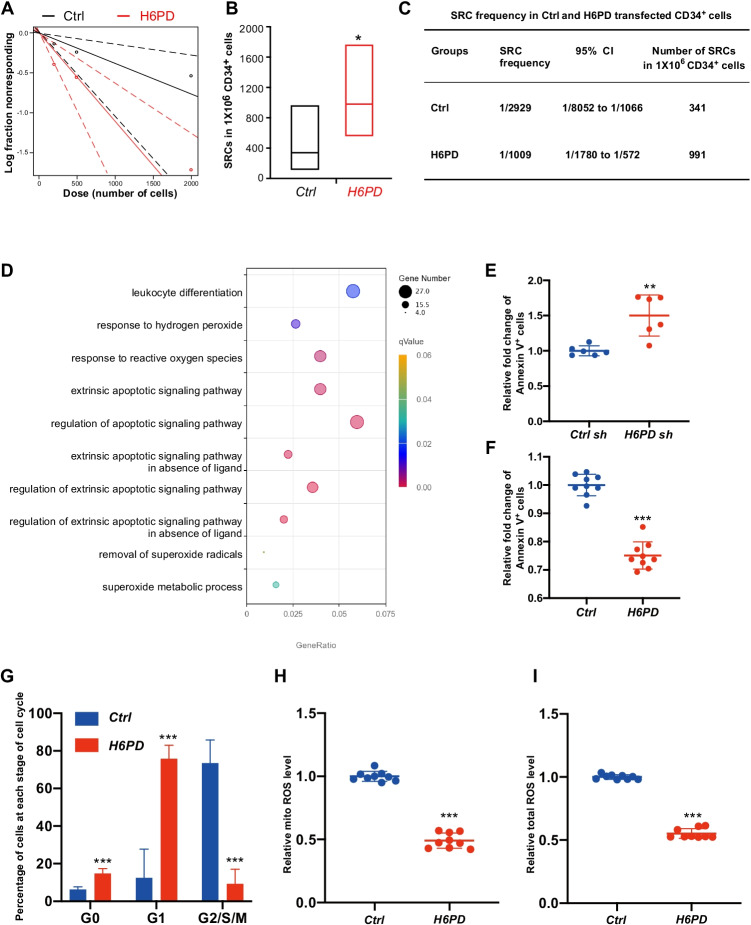
H6PD facilitates expansion of CB HSCs by suppressing ROS generation and cell apoptosis (**A-C**) The frequency of human SRCs in *Ctrl* or *H6PD* transfected CB CD34^+^ cells, as determined by LDA. HSC frequencies (line in the box) and 95% confidence intervals (box) presented as the number of SRCs in 1 × 10^6^ CD34^+^ cells. (*n* = 5–10 mice per group), *p < 0.05. Poisson statistical analysis. (**D**) Dot plot from GO analysis showing ROS and apoptosis related pathways upregulated in *H6PD* knockdown CD34^+^ cells. (**E-F**) Dot plots showing the relative fold change of Annexin V positive CD34^+^ cells by H6PD KD and OE. Data are shown as mean ± s.d.. **p < 0.01. ***p < 0.001. Two-tailed Student’s t-test. (**G**) Statistical data showing the percentage of G0, G1 and G2/S/M cells in Ctrl and H6PD OE CB CD34^+^ cells. Data are shown as mean ± s.d.. ***p < 0.001. Two-tailed Student’s t-test. (**H-I**) Relative mitochondrial (mito) ROS or total ROS level in Ctrl or *H6PD* transfected CB CD34^+^ cells. Data are shown as mean ± s.d.. ***p < 0.001. Two-tailed Student’s t-test

In order to investigate the mechanism by which H6PD regulates CB HSC expansion, we performed RNA-seq analysis with control shRNA vector -transfected CD34^+^ cells and *H6PD* shRNA -transfected CD34^+^ cells. Gene Ontology (GO) analysis shew that pathways involved in superoxide metabolic process, response to reactive oxygen species (ROS) and hydrogen peroxide are significantly activated by loss function of H6PD (Fig. 1D). Besides, mutiple apoptotic signaling pathways are also enriched by *H6PD* KD (Fig. 1D). To confirm the above changes, we analyzed the level of ROS and the percentage of apoptotic cells by both performing *H6PD* OE and KD. *H6PD* KD significantly causes increased apoptosis of CB CD34^+^ cells, while *H6PD* OE largely suppresses apoptosis in ex vivo cultured CB CD34^+^ cells (Fig. 1E-F). We also examined the cell cycle status of control or *H6PD* OE CD34^+^ cells.The percentage of G_0_-stage quiescent cells in H6PD OE group are significantly higher than that of control group (Fig. 1G). *H6PD* OE markedly suppresses accumulation of both mitochondrial ROS and total ROS (Fig. 1H-I). These results demonstrate that H6PD protects CB HSCs from oxidative stress and apoptosis during ex vivo culturing.

The functional HSCs in certain numbers of CD34^+^ cells significantly decrease after ex vivo expansion [5]. Mitochondrial oxidative stress is highly activated upon ex vivo culturing of CB CD34^+^ HSCs and HPC. Under ex vivo expansion induced oxidative stress, functional HSCs were enriched in CD34^+^CD133^+^ADGRG1^+^ population [5]. We found that OE of *H6PD* significantly promoted ex vivo expansion of CD34^+^CD133^+^ADGRG1^+^ HSCs, which was further proved by in vivo transplantation. Mechanistically, ER localized H6PD negatively regulates ROS generation and apoptosis of CB CD34^+^ HSCs and HPCs. *G6PD* OE has no notable effect on ex vivo expansion of CB HSCs and HPCs. It is likely that H6PD governed redox homeostasis may be involved in regulation of CB HSC expansion. Our study suggests that H6PD protects cells from oxidative stress and apoptosis during CB HSC ex vivo expansion, thus providing novel insights into the regulation of cell fitness under ex vivo culture stress.

## Supplementary Information

Below is the link to the electronic supplementary material.

Supplementary file1 (PDF 6850 KB)

Supplementary file2 (PDF 348 KB)

Supplementary file3 (DOCX 16.2 KB)

Supplementary file4 (DOCX 23 KB)

## Data Availability

Raw data will be provided upon reasonable request.
