# Supplementary figures and images for: H6PD overexpression promotes ex vivo expansion of human cord blood hematopoietic stem cells
Source: Stem Cell Rev Rep. 2022 Feb 5;18(5):1878–80. doi: 10.1007/s12015-022-10352-w (PMC9209374; doi:10.1007/s12015-022-10352-w)

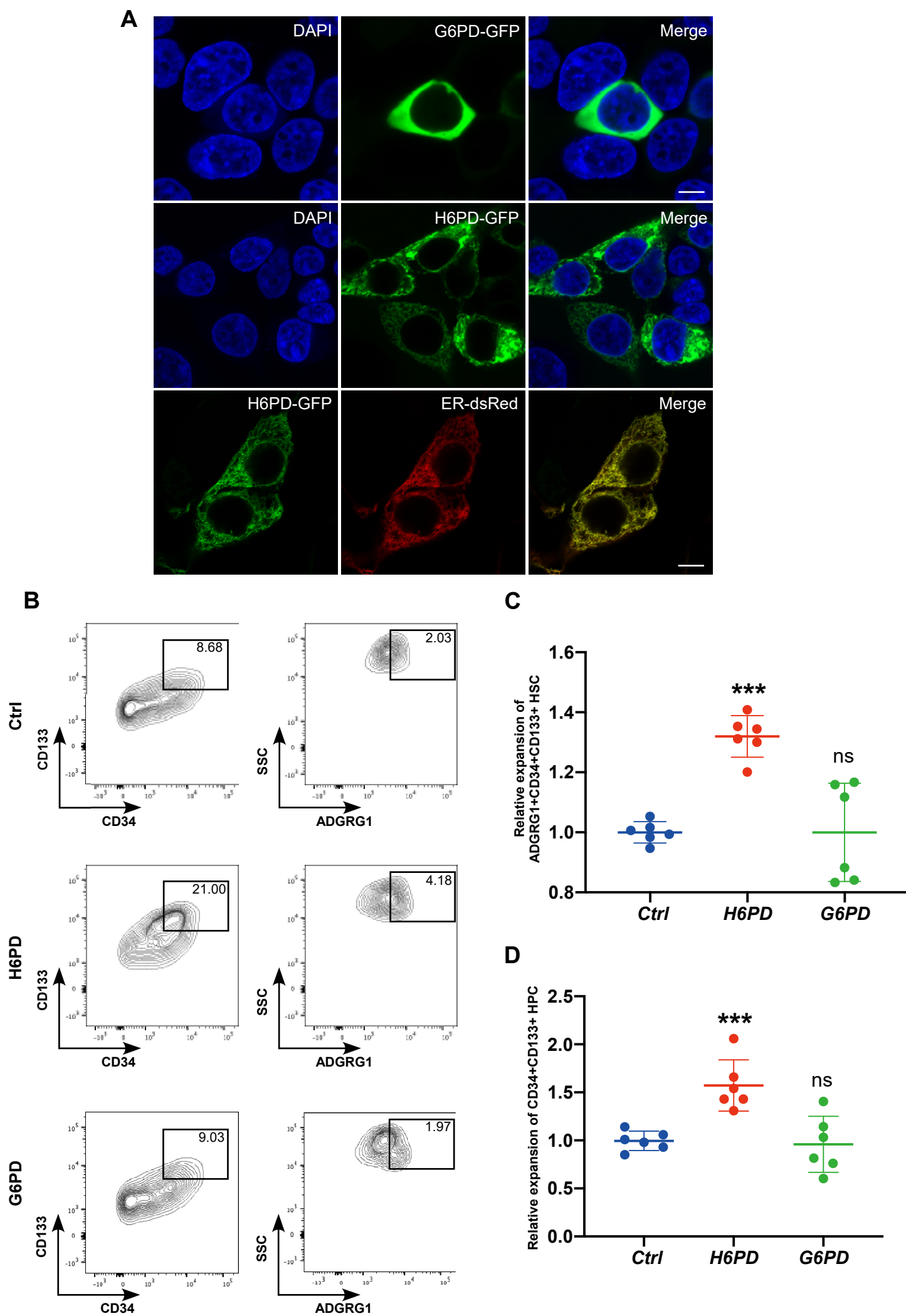

Supplementary Figure 1

Supplement: Supplementary file 1 — Supplementary file1 (PDF 6850 KB) [file 12015_2022_10352_MOESM1_ESM.pdf]

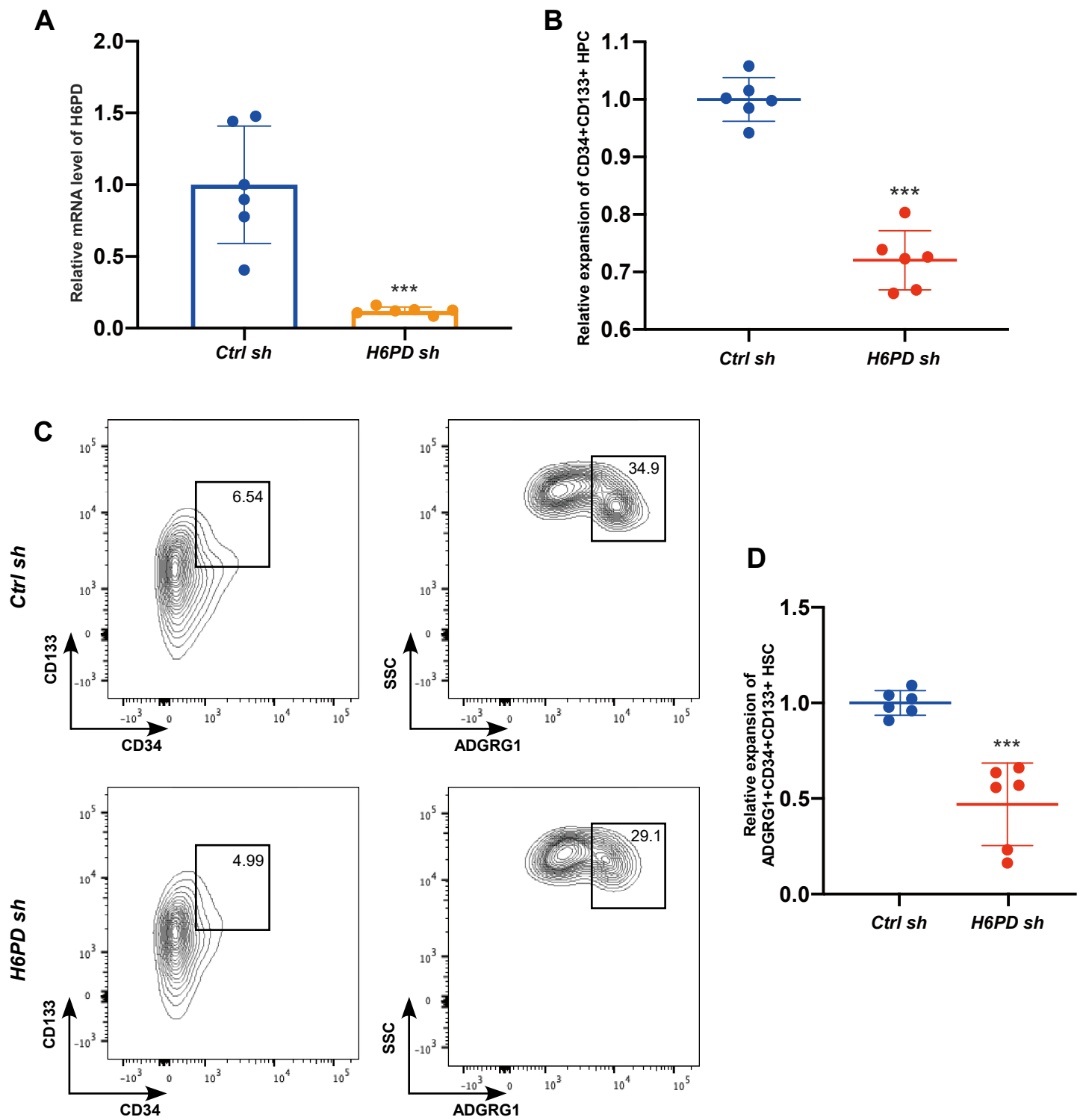

Supplementary Figure 2

Supplement: Supplementary file 2 — Supplementary file2 (PDF 348 KB) [file 12015_2022_10352_MOESM2_ESM.pdf]
