# Supplementary material for: H6PD overexpression promotes ex vivo expansion of human cord blood hematopoietic stem cells
Source: Stem Cell Rev Rep. 2022 Feb 5;18(5):1878–80. doi: 10.1007/s12015-022-10352-w (PMC9209374; doi:10.1007/s12015-022-10352-w)
Supplement: Supplementary file 3 — Supplementary file3 (DOCX 16.2 KB) [file 12015_2022_10352_MOESM3_ESM.docx]

**Supplementary Figure Legends**

**Supplementary Figure 1. Overexpression of *H6PD* but not *G6PD* promotes *ex vivo* expansion of human CB HSCs and HPCs.**

1. Confocal images showing the expression pattern of H6PD and G6PD in 293T cells. DAPI indicates the nucleus. Scale bar: 10 µM.
2. Flow plots showing the frequency of CD34^+^CD133^+^ADGRG1^+^ HSCs in control vector, *H6PD* or *G6PD* transfected CB CD34^+^ cells.
3. Quantification of CD34^+^CD133^+^ADGRG1^+^ cells in control vector, *H6PD* or *G6PD* transfected CB CD34^+^ cells. Data are shown as mean±s.d.. ***p<0.001. ns, not significant. Two-tailed Student’s t-test.
4. Quantification of CD34^+^CD133^+^ cells in control vector, *H6PD* or *G6PD* transfected CB CD34^+^ cells. Data are shown as mean±s.d.. ***p<0.001. ns, not significant. Two-tailed Student’s t-test.

**Supplementary Figure 2. *H6PD* knockdown suppresses *ex vivo* expansion of human CB HSCs and HPCs.**

1. Relative mRNA level of *H6PD* in *Ctrl* shRNA and *H6PD* shRNA transfected 293T cells. ***p<0.001. Two-tailed Student’s t-test.
2. Quantification of CD34^+^CD133^+^ cells in *Ctrl* shRNA and *H6PD* shRNA transfected CB CD34^+^ cells. Data are shown as mean±s.d.. ***p<0.001. Two-tailed Student’s t-test.
3. Flow plots showing the frequency of CD34^+^CD133^+^ADGRG1^+^ HSCs in *Ctrl* shRNA and *H6PD* shRNA transfected CB CD34^+^ cells.
4. Quantification of CD34^+^CD133^+^ADGRG1^+^ cells in *Ctrl* shRNA and *H6PD* shRNA transfected CB CD34^+^ cells. Data are shown as mean±s.d.. ***p<0.001. Two-tailed Student’s t-test.
