# Supplementary material for: H6PD overexpression promotes ex vivo expansion of human cord blood hematopoietic stem cells
Source: Stem Cell Rev Rep. 2022 Feb 5;18(5):1878–80. doi: 10.1007/s12015-022-10352-w (PMC9209374; doi:10.1007/s12015-022-10352-w)
Supplement: Supplementary file 4 — Supplementary file4 (DOCX 23 KB) [file 12015_2022_10352_MOESM4_ESM.docx]

**Methods and Material**

**Animals**

NM-NSG (NOD-PrkdcscidIL2rgem1B2mem1/Smoc) mice (6-8 weeks old) were ordered from Shanghai Model Organisms and maintained in the Laboratory Animal Center of the Shanghai Jiao Tong University School of Medicine (SJTUSM). The protocols of all animal experiments were approved by The Institutional Animal Care and Use Committee of SJTUSM.

**CB CD34^+^ cells isolation and culture**

The isolation and culture of CB CD34+ cells were performed as previously reported[25]. Cord blood samples were provided by Affiliated Sixth People’s Hospital, Shanghai Jiao Tong University. Mononuclear cells were isolated by density gradient centrifugation with Ficoll-Paque Plus (GE Healthcare, Piscataway, NJ, USA). CD34^+^ cells were purified using immunomagnetic selection kit (Miltenyi Biotec, Auburn, CA, USA). CB CD34^+^ cells were cultured in Stemline II medium (Sigma, s0192) with 100 ng/mL stem cell factor (SCF) (R&D Systems, #7466-SC-010/CF), 50 ng/mL thrombopoietin (TPO) (R&D Systems, #288-TP-200/CF), and 50 ng/mL Fms-like tyrosine kinase 3 ligand (FL) (BioLegend, # 710802).

**Immunostaining and Flow cytometry**

Immunostaining and flow cytometry analysis were performed as previously described[12, 25]. Briefly, CD34^+^ Cells were stained with fluorescence conjugated antibodies at 4℃ for 30 min. Cells were washed with cold PBS and cell pellets were fixed with 1% formaldehyde. Samples were analyzed on an LSRII flow cytometer (BD Biosciences). The following antibodies from BD Bioscience (San Jose, CA, USA) were used for cell surface staining (1:200 dilution): CD34-APC (581), CD133-PE-CF594(clone7, BioLegend), ADGRG1-PE (4C3, BioLegend), and CD45-APC (HI30, BD).

**In vivo transplantation**

CD34^+^ CB cells were transfected with control or H6PD vectors and ex vivo cultured for 4 days. Control or H6PD OE cells were intravenously transplanted into sublethally irradiated primary NSG recipient mice (350 cGy; 137^Cs^ source, single dose). The percentage of human CD45^+^ cells in the bone marrow was checked by immuno-staining and flow cytometry at month 4 after transplantation. Recipient mice with more than 0.1% human CD45^+^ cells in the bone marrow were defined as positive transplantation. The HSC frequency was calculated using L-Calc software (Stem Cell Technologies Inc, Vancouver, BC, Canada) and plotted using ELDA software (bioinf.wehi.edu.au/software/elda/).

**RNA sequencing**

Control or H6PD KD CB CD34^+^ cells were lysed and RNA was extracted using RNeasy Mini Kit (QIAGEN, Valencia, CA, USA). The following sequence was used for constructing vector with H6PD shRNA: 5’-ctgatctctaagctggctaat-3’. RNA sequencing analysis was performed at Novogene Co. Ltd. (Tianjin, China).

**Cell Cycle analysis**

CB CD34^+^ cells were fixed and permeabilized with BD Cytoperm^TM^ Permeabilization Buffer Plus (#554714, BD). Wash cells twice with cold PBS containing 1% FBS. Cells were resuspended in 200 μL PBS and incubated with anti-Ki67-Alexa Fluor467(#561126, BD) at room temperature (RT) for 20-30 minutes in the dark. Cells were wash with cold PBS containing 1% FBS and stained with 0.1μg/mL DAPI (#564907, BD). Run FACS immediately to collect the data on a LSRFortessa flow cytometer (BD Biosciences).

**Measurement of Reactive Oxygen Species (ROS)**

For mitochondrial ROS analysis, live CB CD34^+^ cells were incubated with MitoSOX^TM^ Red Mitochondrial Superoxide Indicator (Thermofisher) for 30 minutes at 37°C in the incubator. Cells were washed with ice cold PBS by spinning at 300 g, 4°C, for 10 mins. Remove the supernatant, vortex and resuspend cells in 500 PBS. Run FACS immediately to collect the data on a LSRFortessa flow cytometer (BD Biosciences). For total ROS analysis, live CB CD34^+^ cells were collected by centrifuging at 1500 rpm for 8 min, washed with cold PBS. Cells were stained with the ROS assay loading solution and incubated in a 5% CO2, 37 °C incubator for 1 hour. Samples were analyzed on a LSRFortessa flow cytometer (BD Biosciences).

**Annexin V staining**

Live CB CD34^+^ cells were washed twice with cold PBS, resuspended in 200 μL PBS with 1% BSA. Incubated cells with AnnexinV (#556422, BD) at room temperature (RT) for 20-30 minutes in the dark. Cells were wash with cold PBS and then stained with 0.1μg/mL DAPI (#564907, BD). Run FACS immediately to collect the data on a LSRFortessa flow cytometer (BD Biosciences).

**Statistical analysis**

Statistical analysis was performed using GraphPad Prism 5.0. Data are shown as mean values ± standard deviation (SD). Two-tailed Student’s t-tests were performed for statistical analysis between two groups.
